# Spatial Distribution Patterns and Driving Factors of Plant Biomass and Leaf N, P Stoichiometry on the Loess Plateau of China

**DOI:** 10.3390/plants10112420

**Published:** 2021-11-09

**Authors:** Zhao Fang, Xiaoyu Han, Mingyang Xie, Feng Jiao

**Affiliations:** 1 Institute of Soil and Water Conservation, Northwest A&F University, Xi’an 712100, China; fangzhao16@mails.ucas.ac.cn (Z.F.); 2020060524@nwafu.edu.cn (X.H.); 2 Institute of Soil and Water Conservation, Chinese Academy of Sciences and Ministry of Water Resource, Xi’an 712100, China; Xiemingyang19@mails.ucas.ac.cn

**Keywords:** leaf nitrogen (N) and phosphorus (P) contents, N:P ratio, biomass, herb community, driving factor, Loess Plateau

## Abstract

Understanding the geographic patterns and potential drivers of leaf stoichiometry and plant biomass is critical for modeling the biogeochemical cycling of ecosystems and to forecast the responses of ecosystems to global changes. Therefore, we studied the spatial patterns and potential drivers of leaf stoichiometry and herb biomass from 15 sites spanning from south to north along a 500 km latitudinal gradient of the Loess Plateau. We found that leaf N and P stoichiometry and the biomass of herb plants varied greatly on the Loess Plateau, showing spatial patterns, and there were significant differences among the four vegetation zones. With increasing latitude (decreasing mean annual temperature and decreasing mean precipitation), aboveground and belowground biomass displayed an opening downward parabolic trend, while the root–shoot ratio gradually decreased. Furthermore, there were significant linear relationships between the leaf nitrogen (N) and phosphorus (P) contents and latitude and climate (mean annual rainfall and mean annual temperature). However, the leaf N/P ratio showed no significant latitudinal or climatic trends. Redundancy analysis and stepwise regression analysis revealed herb biomass and leaf N and P contents were strongly related to environmental driving factors (slope, soil P content and latitude, altitude, mean annual rainfall and mean annual temperature). Compared with global scale results, herb plants on the Loess Plateau are characterized by relatively lower biomass, higher N content, lower P content and a higher N/P ratio, and vegetative growth may be more susceptible to P limitation. These findings indicated that the remarkable spatial distribution patterns of leaf N and P stoichiometry and herb biomass were jointly regulated by the climate, soil properties and topographic properties, providing new insights into potential vegetation restoration strategies.

## 1. Introduction

Aboveground biomass (AGB), belowground biomass (BGB) and the ratio of roots to shoots (R/S) are regarded as important parameter of vegetation biomass, playing critical roles in estimating terrestrial ecosystem productivity and in global climate models [1,2,3,4]. Because of the influences of intensive anthropogenic activities, the atmospheric CO_2_ concentration has increased over the past 60 years [5]. Studies have shown that grassland ecosystems account for 1/4 of the Earth’s land surface and 1/10 of global carbon stocks, which fixed the majority of atmospheric CO_2_ [6,7]. Thus, a better understanding of plant biomass is essential for understanding vegetation dynamics, terrestrial ecosystem carbon (C) stocks and their response to environmental changes [8,9,10,11]. Previous studies have demonstrated that plant biomass has important implications for community structure and ecosystem function and is affected by environmental factors [12,13,14]. The varying responses of plant biomass on these environmental factors are complex, and less is known about the interactive effects of potential environmental driving factors (climate, soil properties, topographic properties, etc.) on the herb plant biomass of the Loess Plateau.

Nitrogen (N) and phosphorus (P) are the basic nutrients that limit plant growth [15], and they have close interactive relationships in terrestrial ecosystems [16]. N and P cycles are generally regarded as the main limiting factors for the productivity of an ecosystem, while the ratio of these two elements in leaf tissue may indicate whether a system is limited by N, P, or both [17]. Accordingly, the N/P ratio in plant leaves has become a focal indicator of plant nutrient limitations, adaptation strategies and ecosystem function at the community level and has been studied intensely. In general, an N/P value < 14 indicates that plant growth is mainly limited by N; an N/P value > 16 indicates that plant growth is mainly limited by P, and an N/P value between 14 and 16 indicates that plant growth is limited by both P and N or is not deficient in either nutrient [18]. So far, the increasing efforts are devoted to identifying the geographical pattern of leaf N and P stoichiometry and its relationship with environmental factors at local, regional or global scales [15,19,20,21,22,23]. There are, however, only few studies in Loess Plateau about the geographical pattern of leaf N and P stoichiometry cited in international scientific literatures [24].

The Loess Plateau in China, with an area of 6.2 × 10^5^ km^2^, is a unique area due to its distinctive landscape and deep loess deposits; this area also experiences the most intense soil erosion in the world [25]. In the last century, due to population increases and the intensification of human activities, the Loess Plateau has experienced the most serious vegetation damage and ecological degradation [26]. To improve this dilemma, the Chinese government launched a series of nationwide conservation projects, such as the ‘Grain for Green’ project. The Loess Plateau, with diverse vegetation types and the varying hydrothermal conditions extending from south to north, offers a unique opportunity for determining the critical factors affecting leaf N, P and plant biomass across vegetation transect. However, the knowledge gap regarding the relationships among leaf stoichiometry, plant biomass and vegetation growth in different types of vegetation has not been filled. Changes in leaf stoichiometry and biomass are affected and restricted by various environmental factors, and differences along latitudinal gradients often lead to corresponding changes in environmental factors, such as temperature, light and soil moisture, etc. It is still necessary to study the effects of potential drivers on leaf N and P stoichiometry and herb biomass in different vegetation recovery zones.

In this study, we tested the responses of leaf N and P stoichiometry and plant biomass to latitude and climate in different vegetation zones at fifteen sampling sites spanning from south to north along a 500 km-long latitudinal gradient on the Loess Plateau. Our objectives were to understand the effects of potential drivers (climate, soil properties, topographic properties) on leaf N and P stoichiometry and herb biomass to reveal the limiting conditions of nutrient restrictions, to provide the basis for future ecological restoration and polices on the Loess Plateau and to offer basic data for global scale research.

## 2. Materials and Methods

### 2.1. Study Site

**Figure 1:**
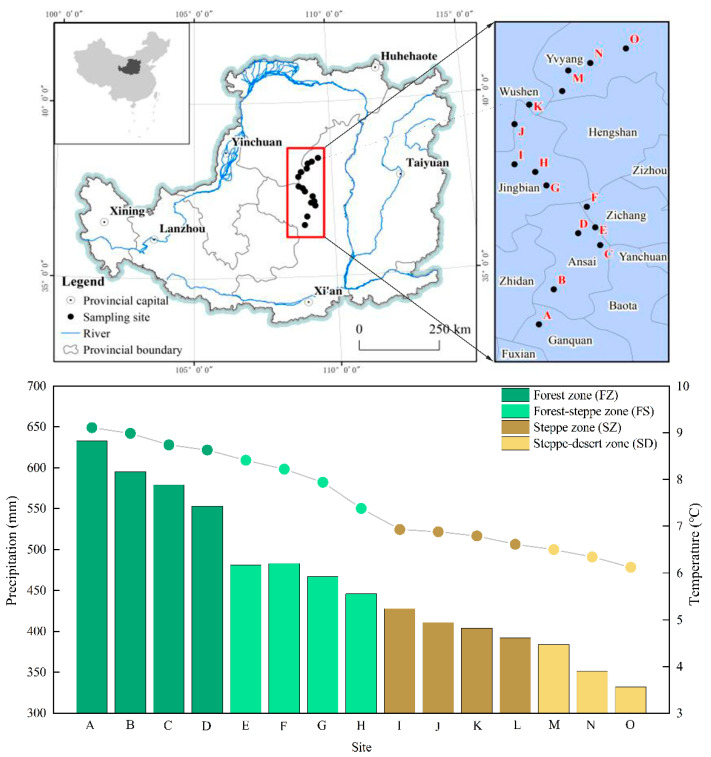
Location of the study area and illustration of sampling sites of mean monthly temperature and precipitation (1990–2010) along a 500-km-long latitudinal gradient on the Loess Plateau. All climate data (MAT and MAP) was obtained from China Meteorological Data Sharing Service System (http://data.cma.cn/ (accessed on 10 May 2021)).

From south to north, the following six representative areas in different vegetation zones on the Loess Plateau were selected as the study areas: Fuxian, Ganquan and Ansai counties in Yan’an city, Jingbian and Hengshan counties and Yuyang district in Yulin city in Shaanxi Province. According to previous studies [27], we established fifteen sampling sites along a 500-km-long latitudinal gradient in the Loess Plateau, which were distributed in forest zone (FZ), forest–steppe zone (FS), steppe zone (SZ) and steppe–desert zone (SD) (Figure 1). The study region was in the midlatitude temperate zone (107°97′–109°87′ E, 35°95′–38°36′ N), with an altitudinal range of about 1015–1600 m above sea level (m.a.s.l), a mean annual temperature (MAT) of 8.8 °C, a mean annual precipitation (MAP) of 505.3 mm, an annual sunshine time of 2395.6 h (the sunshine percentage is 54%) and an annual frost-free period of 157 days. Regional climate conditions, in recent years, have exhibited a warming and wetting trend, with distinct wet and dry seasons for precipitation. The main soil types were loess, loess sandy, and aeolian sandy soils according to the Genetic Soil Classification of China [28]. The land uses include forestland, grassland, and farmland. In this area, the main herb species are *Bothriochloa ischaemum*, *Stipa bungeana*, *Cleistogenes caespitosa*, *Lespedeza davurica*, *Astragalus melilotoides*, *Artemisia sacrorum*, *Heteropappus altaicus* and *Potentilla tanacetifolia*; other concrete description of vegetation and soil conditions can be found in the appendix materials (Table S1).

### 2.2. Experimental Design and Soil Sampling

The vegetation measurements and sampling were conducted in late August 2012, when the vegetation reached maximum biomass and cover. At each site, three sampling plots (1 m × 1 m) with homogeneous vegetation and landform conditions were established to identify all species and investigate the vegetation coverage, height and density. We calculated the community diversity index (included Shannon–Wiener index, Margalef richness index and Pielou evenness index) according to the method of [29]. The AGB of the plants was collected by clipping the plants at ground level. Moreover, all target dominant plant leaves in every quadrat were collected individually to determine the leaf N and P contents at the community level. The BGB (roots < 2 mm in diameter) was collected randomly from the upper 30 cm soil layer with a soil drilling sampling corer (9 cm in diameter). The litter was cleared before sampling. The roots were rinsed with a large amount of deionized water, dried at 70 °C for 48 h to a constant weight and weighed on an analytical balance. The dried leaves were ground to pass through a 0.15 mm sieve for elemental analysis.

Within each quadrat, three soil samples for 0–30 cm were randomly sampled by a soil auger (diameter of 5 cm) and then thoroughly mixed to form one composite sample, and a total of 45 soil samples (3 quadrats × 15 sites) were collected. We patiently removed debris and fine roots by hand when all soil samples were naturally air-dried in the lab, and then they were sieved through 2-mm and 0.15-mm mesh for different element analysis with a ball mill. The total N content of the leaves was measured with a CHNS/O Elemental Analyzer (PekinElmer, Boston, MA, USA), and the total P content was analyzed colorimetrically after H_2_SO_4_-H_2_O_2_-HF digestion using the molybdate/stannous chloride method [30]. Soil organic C and total N and P contents were determined using standard testing methods as described by Jiao et al. [31]. The contents of nutrient contents were expressed as mg·g^−1^ on a dry mass basis.

### 2.3. Data Analysis

Data, including all tables and figures, are presented as mean ± standard deviation (SD). A linear mixed-effect model (LMM) with Tukey’s multiple comparisons test (*p* < 0.05) was conducted to assess the differences of leaf N, P stoichiometry and biomass among four vegetation zones in SPSS 22.0. The “vegetation zone” was used as the fixed factor and “sampling site” was used as a random factor. Linear or curvilinear (quadratic) regressions were adopted to explore the relationships of independent variables (MAT, MAP and latitude) with the dependent variables (leaf N content, leaf P content, leaf N:P ratio, AGB, BGB, and R/S ratio). All data were checked for normality and homogeneity of variance before conducting the parametric tests. Redundancy analysis (RDA) and stepwise regression analysis (SRA) were performed to identify the critical factors of herb biomass and leaf N and P stoichiometry. RDA was conducted in Canoco 5.0, and all figures were created in Origin 2018.

## 3. Results

### 3.1. Spatial Variation of Plant Biomass and Leaf N, P Stoichiometry along the Latitude Gradient

**Figure 2:**
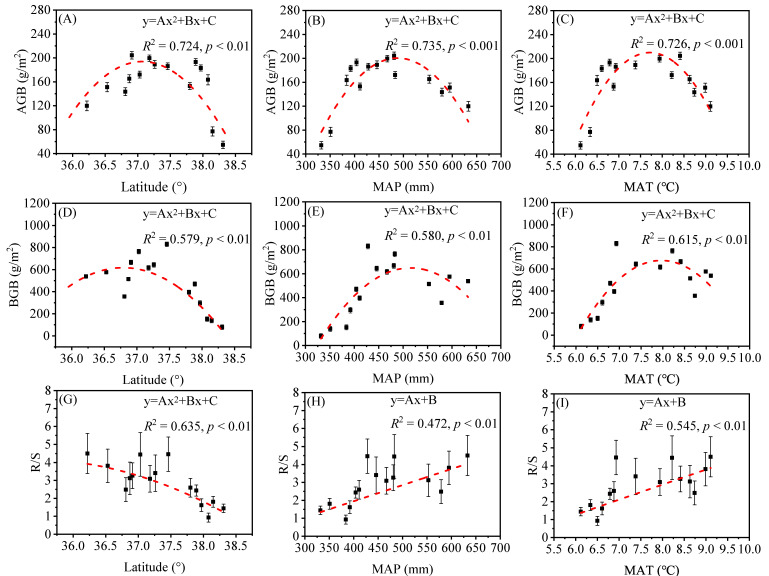
Relationships of AGB, BGB and R/S with MAT, MAP and absolute latitude. Note: (**A**–**C**), AGB: above-ground biomass; (**D**–**F**), BGB: below-ground biomass; (**G**–**I**), R/S: root-to-shoot ratio. The red lines indicate the fits of the linear model of AGB, BGB and R/S and environmental gradient (latitude, MAT and MAP). All climate data (MAT and MAP) was obtained from China Meteorological Data Sharing Service System (http://data.cma.cn/ (accessed on 10 May 2021)).

**Figure 3:**
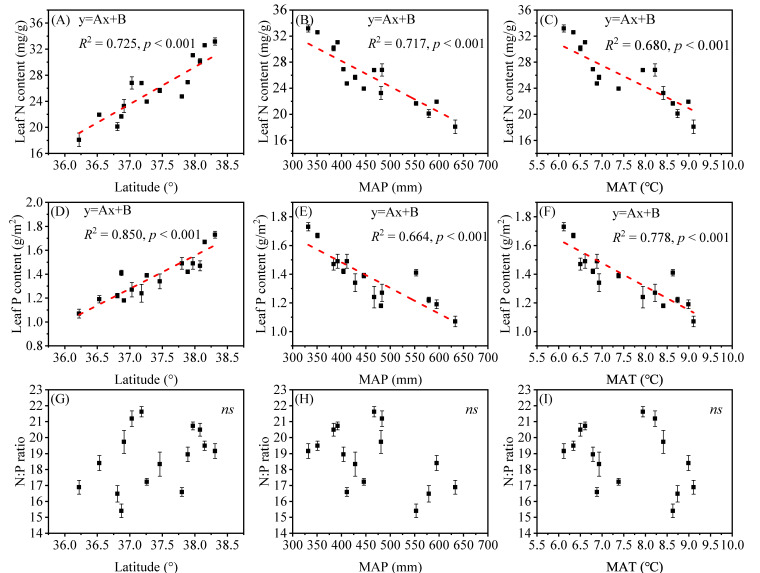
Linear regression relationships of Leaf N, P and the leaf N/P ratio with MAT, MAP and absolute latitude. Note: (**A**–**C**), Leaf N: leaf nitrogen content; (**D**–**F**), Leaf P: Leaf phosphorus content; (**G**–**I**), N:P ratio: Leaf N/P ratio. The red lines indicate the fits of the linear model of Leaf N, P and the leaf N/P ratio and environmental gradient (latitude, MAT and MAP). All climate data (MAT and MAP) was obtained from China Meteorological Data Sharing Service System (http://data.cma.cn/ (accessed on 10 May 2021)).

Plant biomass of the herb communities on the Loess Plateau across of the sampling sites ranged from 54.60 to 204.32 g/m^2^ (CV = 27.8%) for AGB, 78.88 to 829.64 g/m^2^ (CV = 48.8%) for BGB and 0.93 to 4.50 (CV = 13.5%) for R/S. As shown in Figure 2, with increasing latitude (decreasing mean annual temperature, MAT and decreasing mean precipitation, MAP), AGB and BGB displayed an opening downward parabolic trend (Figure 2A–F). However, the root–shoot ratio (R/S) decreased linearly with increasing latitude (decreasing MAT and decreasing MAP) (Figure 2G–I). By contrast, leaf N and P stoichiometry of herbs on the Loess Plateau across the sampling sites ranged from 20.45 to 31.96 mg/g (CV = 17.1%) for leaf N content, 1.22 to 1.62 mg/g (CV = 13.9%) for leaf P content and 16.90 to 19.94 (CV = 9.94%) for the leaf N/P ratio. The mean leaf N, P and N/P values were 25.79 mg/g, 1.37 mg/g and 18.71, respectively. Leaf N and P contents exhibited significant relations to latitude, MAT and MAP, but not N/P ratio. Besides, linear regression showed that the leaf N and P contents were substantially correlated with the latitude and increased with increasing latitude (decreasing MAT and decreasing MAP). However, the leaf N/P ratio was not positively correlated with environmental variables (latitude, MAT and MAP) (Figure 3).

### 3.2. Differences in Herb Biomass and Leaf N, P Stoichiometry among Vegetation Types

**Figure 4:**
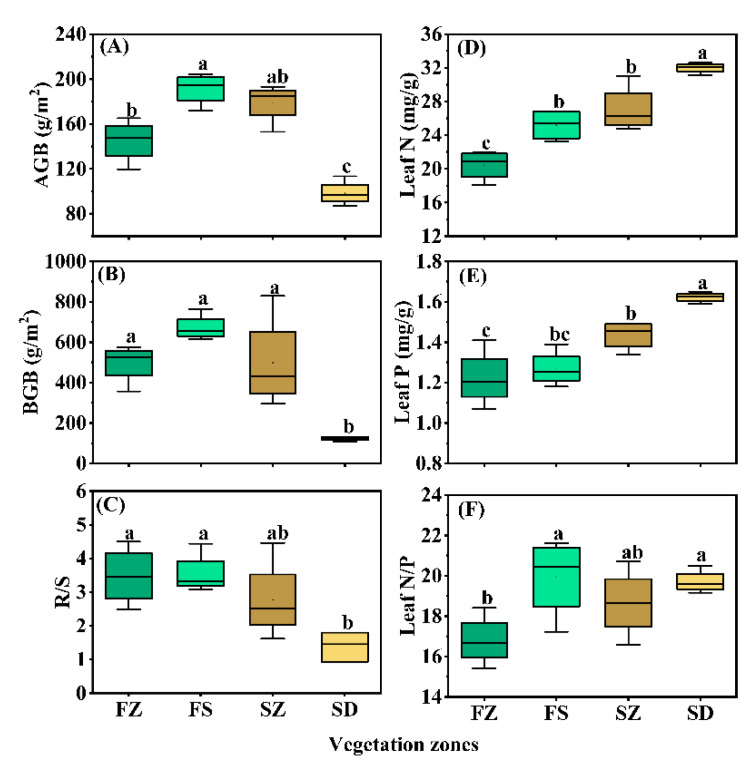
Boxplots of leaf N, P stoichiometry and plant biomass in each of vegetation zones along latitudinal gradients. Note: (**A**), AGB: above-ground biomass; (**B**), BGB: below-ground biomass; (**C**), R/S: root-to-shoot ratio; (**C**,**F**), FZ: forest zone; FS: forest–steppe zone; SZ: steppe zone; SD: steppe–desert zone. (**D**), Leaf N: leaf N content; (**E**), Leaf P: leaf P content. The boundaries of the box represent the lower (25%) and upper (75%) quartiles of data, respectively, and the median is represented by the line inside the box. Different lowercase letters above the bars indicate significant differences (ANOVA, *p* < 0.05) among different vegetation zones.

To reveal the differences in herb biomass between different vegetation types in the Loess Plateau, the results of LMM showed that AGB, BGB and R/S of the steppe-desert zone were significantly lower than those in the other three vegetation zones (Figure 4A–C and Table S4) (*p* < 0.05). The biomass order of the herb communities (AGB, BGB) was FS > SZ > FZ > SD, while the root–shoot ratio (R/S) ranked as FS > FZ > SZ > SD. The biomass allocation of FZ, FS and SZ was predominantly concentrated in the belowground part (2 to 4 times), except for SD. Additionally, we compared the differences in the leaf N and P contents and N/P ratio among the different vegetation zones and found that the plant leaf N and P contents in the steppe–desert were significantly higher than those in the other three vegetation zones (Figure 4D,E and Table S4), and the lowest leaf N/P ratio was found in the forest zone (*p* < 0.05) (Figure 4F and Table S4).

### 3.3. Factors Driving Herb Biomass and Leaf N, P Stoichiometry

**Figure 5:**
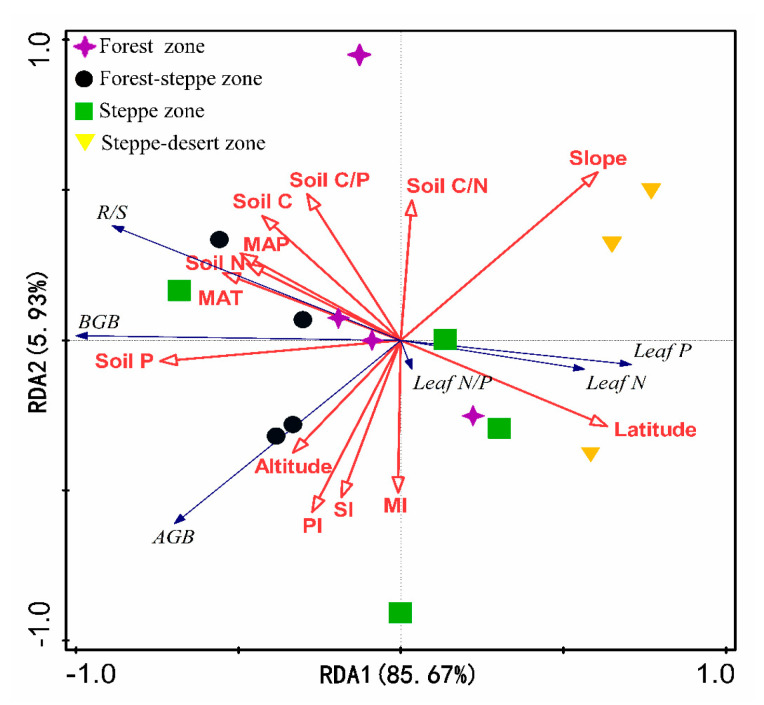
Redundancy analysis (RDA) of the relationship between plant biomass, leaf N and P stoichiometry and environmental driving factors in different vegetation zones. Note: AGB, above-ground biomass; BGB, below-ground biomass; R/S: root-to-shoot ratio; Leaf N: leaf nitrogen content; Leaf P: leaf phosphorus content; Leaf N/P: leaf nitrogen/ phosphorus ratios; Soil C: soil organic carbon; Soil N: soil total nitrogen; Soil P: soil total phosphorus; Soil C/N: soil carbon/nitrogen ratios; Soil C/P: soil carbon/ phosphorus ratios; Soil N/P: soil nitrogen/ phosphorus; MAP: mean annual precipitation; MAT: mean annual temperature; SI: Shannon–Wiener diversity index; MI: Margalef richness index; PI: Pielou evenness index. All climate data (MAT and MAP) was obtained from China Meteorological Data Sharing Service System (http://data.cma.cn/ (accessed on 10 May 2021)).

RDA analysis showed that plant biomass and leaf N and P stoichiometry of herbs were determined by topographic properties, soil C:N:P stoichiometry and climatic conditions. A total of 85.67% and 5.93% were accounted for by the first two axes, respectively (Figure 5). Topographic properties and species diversity were strongly correlated with AGB, BGB and R/S. The BGB, R/S, and leaf N and P contents were mainly correlated to the soil C:N:P stoichiometry and climatic conditions, while the N/P ratio of leaves was less affected by the above factors. Moreover, we performed a stepwise regression to detect the critical factors of topographic properties, soil C:N:P stoichiometry and climatic conditions that affected plant biomass and leaf N and P stoichiometry. Taken together, these results demonstrate that the slope; soil P content; latitude, altitude and MAT; MAP and latitude were the key driving factors of AGB, BGB, R/S, leaf N and P, respectively. However, no critical factor had a strong effect on leaf N/P (Table 1).

## 4. Discussion

### 4.1. The Spatial Patterns of Herb Biomass and Response to Environmental Factors

On the Loess Plateau, precipitation and temperature decrease toward the northwest with increasing latitude (Figure 1), and therefore, the vegetation zones change from forest to desert with increasing latitude from southeast to northwest [27]. Differences in environmental conditions (temperature, precipitation, etc.) are often linked to varying latitudes and, in turn, could be used to explain the distribution of vegetation types across the Loess Plateau. The results from our study suggest that the biomasses of the herbaceous communities in the different vegetation zones in the loess hilly region were in the order of forest–steppe zone > grassland zone > forest zone > steppe–desert zone, and the variations in AGB and BGB with increasing latitude (decreasing mean annual temperature, MAT and decreasing mean precipitation, MAP) showed an opening downward parabolic trend (Figure 2). Besides, compared with other temperate grasslands in the world [3,32], the AGB, BGB and R/S ratio of the Loess Plateau were relatively lower but only slightly higher than China [33] and the Inner Mongolia grassland [34] (Table S2). Hydrothermal factors are important factors that restrict the biomass of herbaceous communities in semiarid areas [35,36], which contributed to the lower biomass of herbaceous vegetation on the Loess Plateau. The most common environmental stressor affecting plant growth in arid and semiarid regions is an insufficient water supply [37]. To better adapt to arid environments, the plant might allocate more resources to root growth to obtain more nutrients and water [12,38]. Changes in precipitation may shape the various physiological traits of plants by affecting the moisture regimes in arid and semiarid regions [39,40]. Thus, decreasing precipitation with increasing latitude will change the soil water availability [41] and indirectly affect the herbaceous biomass. Besides, temperature may influence various metabolic processes in plants by affecting the activities of enzymes, such as water and mineral absorption, material synthesis, transformation, transportation, and distribution, and further affecting the function of cells, thereby affecting the normal metabolic activities of plants. On the other hand, aggravated local water scarcity after afforestation and soil evaporation caused by increasing temperatures may be unfavorable for the growth of herbaceous vegetation in forest zones [42]. The steppe–desert zone and steppe zone have relatively less rainfall and lower temperatures; herbaceous plant growth is affected by hydrothermal factors, thus restraining plant growth, resulting in the lower AGB and BGB of the herbaceous community. However, with increasing latitude, precipitation increases improve soil water availability, and large amounts of litter accumulate in the forest zone and forest–steppe zone; these two factors jointly regulate soil nutrient dynamics via decomposition and element release from surface litter. Compared with SZ and SD, soil nutrients and hydrothermal conditions in FS are more conducive to the growth of herbaceous vegetation, thus showing the highest AGB and BGB.

RDA and SRA revealed herb biomass were strongly related to environmental driving factors, among them the slope, soil P content and latitude, altitude, MAT, which were key factors affecting AGB, BGB and R/S in the Loess Plateau, respectively. It is well known that the productivity of grasslands is mainly affected by soil water availability rather than directly by rainfall [37]. The slope is believed to play an important role in the distribution of soil moisture, which will significantly affect the productivity and vegetation patterns of grasslands [43,44]. Soil P content, as a key factor affecting belowground biomass, is related to the obvious P limitation of herbaceous vegetation on the Loess Plateau [45]. The difference in environmental driving factors of AGB and BGB causes the R/S to be affected by multiple environmental factors (latitude, altitude, MAT).

### 4.2. The Spatial Patterns of Leaf N and P Stoichiometry and Response to Environmental Factors

Confirming the results of previous studies [20,22], the leaf N and P contents increased with increasing latitude (decreasing MAT and decreasing MAP), which is a phenomenon that can be explained well by the temperature-plant physiological hypothesis [21]. Besides, our research also reveals that MAP and latitude were the key driving factors of leaf N and P, respectively. This is mainly because herb plants usually absorb highly mobile available nitrogen (such as nitrate nitrogen and ammonia nitrogen), but higher rainfall easily causes these highly mobile nitrogens to be leached, making the available nitrogen that can be absorbed and utilized decreases and resulting in a decrease in the N content of leaves [46].

With increasing latitude, the hydrothermal conditions change, and low temperatures affect the RNA efficiency of N- and P-rich enzymes, which reduces the plant biochemical reaction rate; however, increases in leaf N and P contents can compensate for the loss caused by the lower biochemical reaction rate [21]. Therefore, plants need to maintain high leaf N and P levels to offset the low temperature-induced inhibition of metabolic reactions, thus representing an adaptation of plant tissues to low temperature and environmental changes [21]. However, the correlation between N:P and latitude was not significant. On the one hand, our study area represented a relatively small latitude range; on the other hand, the focus of our study was limited to herbal communities. Both factors contributed to the leaf N:P characteristics, and the results were not consistent with those of previous studies [21,47].

To determine the leaf nutrient levels and leaf N/P characteristics of the herbaceous vegetation on the Loess Plateau in depth, we compared the results of this study with other scholars’ findings; we found that the leaf N concentrations of herbaceous plants on the Loess Plateau were significantly higher than the level at the global scale [20,22,48] but were also slightly higher than that found on the Loess Plateau [24]. Unlike previous research [20,22,48], our research object was only limited to herbs and the sample size was relatively small, which may account for the discrepancy in our study.

Furthermore, the leaf P content on the Loess Plateau was significantly lower than that at the global scale [20,22,48] but was also slightly lower than on the Loess Plateau [24] and throughout China [22]. The N/P threshold is often used as an indicator of relative N and P restriction [17,18,19,49]. Compared with the results obtained in studies at the global scale [21], the higher N/P and lower leaf P contents of plants on the Loess Plateau [24] and throughout China [22,23] further indicate that the growth of plants in China is more restricted by P (Table S3).

## 5. Conclusions

In summary, we examined the spatial patterns of herb biomass and leaf N and P stoichiometry and their influencing factors on the regional scale, providing important information about better vegetation management and restoration on the Loess Plateau, and supplementing basic data for global scale research. Our results suggested that the leaf N and P stoichiometry and herb biomass on the Loess Plateau showed remarkable spatial distribution patterns and were regulated by the climate, soil properties and topographic properties. Compared with global scale results, the vegetative growth of the herb community on the Loess Plateau is more susceptible to P limitation. Further, more future research should focus on exploring the driving mechanisms of vegetation dynamics under increasing warming and wetting trend of the Loess Plateau.

## Figures and Tables

**Table 1:** Stepwise regression analysis (SRA) used to identify the key factors of plant biomass and leaf N and P stoichiometry.

Variables	Equations	R^2^	Sig.
AGB	AGB = −4.929SLO + 243.061	0.764	0.001 **
BGB	BGB = 722.128SOP − 107.608	0.740	0.002 **
R/S	R/S = −4.025LAT + 0.02ALT − 1.518MAT	0.927	0.000 **
Leaf N	Leaf N = − 0.045MAP + 46.653	0.915	0.000 **
Leaf P	Leaf P = 0.258LAT − 8.279	0.905	0.000 **
Leaf N/P	—	—	—

AGB, above-ground biomass; BGB, below-ground biomass; R/S, root-to-shoot ratio; SLO, slope; SOP, Soil P content; LAT, latitude; ALT, altitude; MAT, mean annual temperature; MAP, mean annual precipitation (** *p* < 0.01;). All climate data (MAT and MAP) was obtained from China Meteorological Data Sharing Service System (http://data.cma.cn/ (accessed on 10 May 2021)).

## Data Availability

All available data can be obtained by contacting the corresponding author.

## Supplementary Materials

The following are available online at https://www.mdpi.com/article/10.3390/plants10112420/s1, Table S1. Basic information of the experimental plots; Table S2. Comparisons of AGB, BGB and R/S of Loess Plateau with other temperate grasslands in the world. Table S3. Comparisons of leaf N and P stoichiometry around the world. Table S4. Linear mixed-effect model showing the differences in herb biomass and leaf N and P stoichiometry on the Loess Plateau.
