# Supplementary material for: Spatial Distribution Patterns and Driving Factors of Plant Biomass and Leaf N, P Stoichiometry on the Loess Plateau of China
Source: Plants (Basel). 2021 Nov 9;10(11):2420. doi: 10.3390/plants10112420 (PMC8625930; doi:10.3390/plants10112420)
Supplement: Supplementary file 1 [file plants-10-02420-s001.zip › plants-1426824-supplementary.pdf]

Supplementary files

Table S1. Basic information of the experimental plots.

Vegetation zone	Study site	MAT (℃)	MAP (mm)	Slope (°)	Altitude (m)	Soil type	Main herbaceous species
FZ	A	9.11	633	28	1100	I	*Stipabungeana, Rubia cordifolia, Phragmites australis, Taraxacum mongolicum*
	B	8.99	595	17	1105	I	*Stipabungeana, Dracocephalum moldavica, Patrinia scabiosaefolia, Phragmites australis*
	C	8.74	579	13	1015	I	*Stipabungeana, Lespedeza davurica, Taraxacum mongolicum, Incarvillea sinensis*
	D	8.63	553	21	1100	I	*Stipabungeana, Lespedeza davurica, Taraxacum mongolicum, Phragmites australis*
FS	E	8.41	481	19	1300	I	*Stipabungeana, Melilotus officinalis, Artemisia gmelinii, Lespedeza davurica*
	F	8.22	483	11	1330	I	*Stipabungeana, Melilotus officinalis, Saussurea amurensis, Lespedeza davurica*
	G	7.94	467	15	1300	I	*Lespedeza davurica, Artemisia gmelinii, Bidens pilosa*
	H	7.38	446	8	1277	II	*Artemisia gmelinii, Phragmites australis, Stipabungeana, Heteropappus altaicus*
SZ	I	6.93	428	11	1500	II	*Stipabungeana, Artemisia capillary, Dracocephalum moldavica, Lespedeza davurica*
	J	6.88	411	16	1500	II	*Stipabungeana, Lespedeza davurica, Artemisia desertorum, Scorzoneradivaricata*
	K	6.79	404	12	1600	II	*Polygala tenuifolia, Erodium stephanianum, Dracocephalum moldavica, Vicia sepium*
	L	6.61	392	12	1400	II	*Artemisia ordosica, Stipabungeana, Setaria viridis, Artemisia scoparia*
SD	M	6.5	384	23	1100	III	*Artemisia desertorum, Heteropappus altaicus, Artemisia scoparia, Lespedeza davurica*
	N	6.34	351	27	1148	III	*Artemisia desertorum, Artemisia scoparia, Astragalus adsurgens*
	O	6.12	332	29	1205	III	*Artemisia desertorum, Artemisia scoparia, Lespedeza davurica*

[S1] FZ, Forest zone; FS, Forest-steppe zone; SZ, Steppe-zone; SD, Steppe-desert zone; MAT, mean annual temperature; MAP, mean annual precipitation; Ⅰ, Loessial soil, Ⅱ, Loess sandy soil; III, Aeolian sandy soil.

Table S2. Comparisons of AGB, BGB and R/S of Loess Plateau with other temperate grasslands in the world.

Region/temperate grassland	Biomass ($g \cdot m^{-2}$)		R/S			Reference
	AGB	BGB	Mean	Median	Range	
Global	430.2	1810.9	4.50	4.20	1.59～9.90	[3]
Europe	377.00	1903.08	3.70	3.40	1.10～6.90	[32]
North America	207.80	1469.60	4.40	3.70	1.20～10.30	[32]
Japan	742.00	1415.10	4.30	4.30	1.60～6.90	[32]
China	104.80	570.20	6.30	5.70	0.40～14.30	[33]
Inner Mongolia	135.30	775.20	—	—	—	[34]
Loess Plateau	148.98	469.20	2.89	3.09	0.93～4.50	This study

Table S3. Comparisons of leaf N, P stoichiometry around the world.

Region	Leaf N		Leaf P		Leaf N/P		Reference
	Mean	CV	Mean	CV	Mean	CV	
Global	20.09	0.43	1.77	0.63	13.80	0.68	[21]
China	19.09	0.42	1.56	0.74	15.39	0.54	[23]
China	20.20	0.42	1.45	0.99	16.30	0.57	[22]
Loess Plateau	24.10	0.35	1.60	0.34	15.40	0.26	[24]
Loess Plateau	25.79	0.17	1.37	0.13	18.71	0.10	This study

Table S4. Linear mixed-effect model showing the differences in herb biomass and leaf N, P stoichiometry on the Loess Plateau.

Items	Fixed effect (Vegetation type)		Random effect (Sampling site)
	$t_{1,24}$	p-value	SD
AGB (g/m^2)	**4.348**	**< 0.001**	0.458
BGB (g/m^2)	**8.246**	**< 0.001**	0.017
R/S	**2.840**	**0.009**	0.036
Leaf N (mg/g)	**16.049**	**< 0.001**	0.146
Leaf P (mg/g)	**6.630**	**< 0.001**	0.613
Leaf N/P	−0.645	0.525	0.118

S4 Bold indicates statistical significance at $p < 0.05$. AGB represents above-ground biomass, BGB, below-ground biomass; R/S, root to shoot ratio; Leaf N, leaf N content; Leaf P, leaf P content; Leaf N/P, leaf N/P ratio.
